# *Glossina pallidipes* Density and Trypanosome Infection Rate in Arba Minch Zuria District of Gamo Zone, Southern Ethiopia

**DOI:** 10.1155/2022/3004054

**Published:** 2022-10-22

**Authors:** Ephrem Tora, Wasihun Seyoum, Firew Lejebo

**Affiliations:** ^1^Department of Animal Science, College of Agricultural Sciences, Arba Minch University, Arba Minch, Ethiopia; ^2^National Institute for Control and Eradication of Tsetse Fly and Trypanosomosis, Arba Minch, Ethiopia

## Abstract

**Background:**

African trypanosomosis is a disease of both animals and humans resulting from infection with parasitaemic protozoa of the genus Trypanosoma transmitted mainly by the tsetse flies (Glossina species). The disease has been reported in different parts of the country. However, information on the apparent density and trypanosome infection rates of the vectors is very limited in the Southern part of Ethiopia. Therefore, this study was conducted to estimate the apparent density, infection rate of trypanosomes in *Glossina pallidipes*, and the trypanosome species involved in Arba Minch Zuria district of Southern Ethiopia.

**Methods:**

A cross-sectional study was conducted from January to June 2018 in two purposely selected kebeles of Arbaminch Zuria district and in the escarpments of Nech Sar National Park of Southern Ethiopia. For entomological survey, a total of 40 standard NGU traps were deployed around the watering and grazing areas. A total of 300 fresh *Glossina pallidipes* were examined for trypanosome infection using a dissection procedure as described by the FAO Training manual for tsetse control personnel.

**Results:**

The study revealed the presence of only one Glossina species, known as *Glossina pallidipes*, and biting flies including *Stomoxys* and *Tabanus*. A total of 2176 flies were caught of which 1803 (82.86%) belong to *Glossina pallidipes* and the remaining 373 (17.14%) were biting flies. The overall apparent density of *Glossina pallidipes* and biting flies in the study area were 15.03 fly/trap/day (F/T/D) and 3.11 F/T/D, respectively. Relatively higher *Glossina pallidipes* and biting flies, respectively, were caught in a wood-grass land (15.87 F/T/D and 3.69 F/T/D) and riverine forest (15.13 F/T/D and 3.42 F/T/D) than bush land vegetation types (13.87 F/T/D and 1.76 F/T/D). The overall trypanosome infection rate of *Glossina pallidipes* was 17.67% (53/300). Two trypanosome species, namely, *Trypanosoma congolense* (66.04%) and *Trypanosoma vivax* (33.96%), were responsible for *Glossina pallidipes* infection in the study area. Trypanosome infection rate was significantly higher in female *G. pallidipes* than in male (OR = 3.39, 95%CI = 1.53‐7.52). Significantly, higher trypanosome infection rate was observed in flies older than 20 days (OR = 2.5, 95%CI = 1.12‐5.56) and in hunger stage 1 flies (OR = 14.24, 95%CI = 4.01‐50.59). *Glossina pallidipes* infection was significantly higher in and around park grazing areas (OR = 3.41, 95%CI = 1.76‐6.6) and wood-grass land vegetation type (OR = 2.7, 95%CI = 1.2‐6.5).

**Conclusion:**

The current study revealed high apparent density and trypanosome infection in *Glossina pallidipes* in Arba Minch Zuria district of Southern Ethiopia. So, this study warrants the need for strengthening both vector and parasite control strategies in the study area.

## 1. Introduction

Ethiopia has a huge and diverse livestock population in Africa that plays an important role in the national economies and livelihoods of farmers and pastoralists [1]. The livestock subsector contributes about 16.5% of the national gross domestic product (GDP) and 35.6% of the agricultural GDP [2, 3]. It serves as a “living account” or “living bank” for rural and urban poor livestock owners or farmers of the country. Among diverse livestock population, cattle are the primary resource for people in Ethiopia [2–4]. Despite the large cattle population, productivity in Ethiopia is too low and even below the average for most of Eastern and sub-Saharan African countries, due to a number of complex and interrelated factors such as widespread diseases, inadequate feed and nutrition, poor genetic potential of local breeds, and inefficiency of livestock development services [3]. Among the constraints associated with animal health, tsetse-transmitted trypanosomosis is one of the major health factors for the low livestock production and agricultural development [4].

Tsetse-transmitted trypanosomosis is a chronic haemoprotozoan disease of domestic animals and humans caused by different species of unicellular eukaryotic parasite of the genus *Trypanosoma*. With a single exception of *Trypanosoma equiperdum* of equines which is a venereal disease, all of those remaining have arthropod vectors in which transmission is either cyclically by tsetse flies of *Glossina* species or noncyclical (mechanically) by many other biting flies and insects [5, 6]. Domestic animals infected with tsetse-transmitted trypanosomosis can show major clinical manifestations such as anaemia, intermittent fever, anorexia, apathetic, dullness, watery ocular discharge, superficial lymph nodes enlargement, and reproductive disorder. The animals progressively become emaciated and cachectic and finally die if untreated [7].

Tsetse-transmitted bovine trypanosomosis is highly prevalent and distributed in the most arable and fertile land of southwest and northwest parts of Ethiopia following the low lands and greater river basins of Ghibe, Omo, Abay, Akabo, Didessa, and Baro [8, 9]. The disease has been reported in different parts of the country with apparent prevalence ranging from 1.38 to 17.15% [10]. Currently, about 220,000 km^2^ of the above-mentioned regions of the country are infested by five Glossina species, namely, *G. pallidipes*, *G. morsitans submorsitans*, *G. fuscipes fuscipes*, *G. tachinoides*, and *G. longipennis* [11–13]. In the country, the most commonly reported and important *trypanosoma* species affecting cattle include *T. congolense*, *T. vivax*, and *T. brucei* [11, 12]. It is estimated that 10 to 14 million cattle heads in Ethiopia are exposed to the risk of trypanosomosis [9]. Cattle plays a key role in the livelihood of communities of Southern regions of Ethiopia but their production potential is not fully utilized due to tsetse transmitted bovine trypanosomosis [11, 14].

Arba Minch Zuria district of Southern Ethiopia is one of the well-known tsetse belts of East Africa. The district is highly infested with *Glossina pallidipes* and biting flies like *Tabanus* and *Stomoxys* [13, 15–17]. In turn, bovine trypanosomosis is one of the most important livestock diseases in the district, which poses a serious threat to the lives and livelihood of entire communities. Almost all cattle in and adjacent to the district are at risk of acquiring the disease at any time. As a result, people in the district suffer from low level of draught power and productivity of their animals [13, 17–19].

For over the past few decades, many efforts have been made to control tsetse and trypanosomosis in Ethiopia through coordinated action of the government, nongovernmental organizations, and local community. The control interventions commonly used in Ethiopia include insecticidal pour-on, insecticide impregnated traps and targets, and use of different trypanocidal drugs [4, 12, 20]. However, information related to infection rates of trypanosomes in Glossina species, their distribution, magnitude, and involved trypanosome species remains very limited and may be a reason that control strategies are less effective and failed in endemic areas [14, 21]. Hence, the epidemiological knowledge on the infection rate and distribution of tsetse fly are paramount in formulating appropriate strategies for the control of these problems [20, 22]. In our current study area, no studies have been conducted regarding trypanosome infection rate in tsetse fly and its apparent density. The objective of this investigation was to estimate the infection rate of trypanosomes in *Glossina pallidipes*, their apparent density, and involved trypanosome species in Arbaminch Zuria district of Gamo Zone, Southern Ethiopia.

## 2. Materials and Methods

### 2.1. Study Area Description

**Figure 1:**
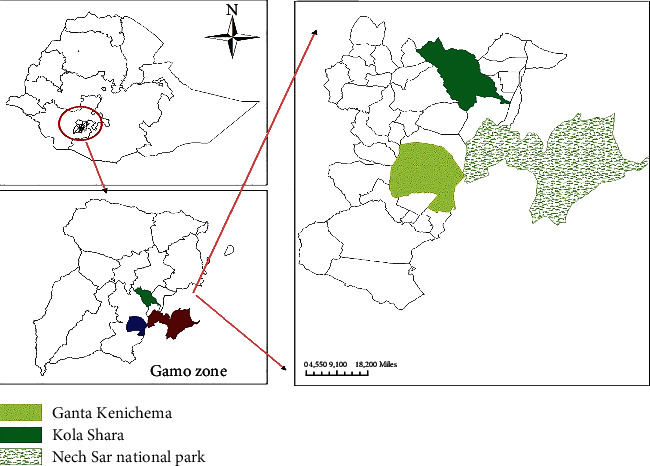
Map of the study area.

The study was conducted in Arba Minch Zuria district of Gamo zone, Southern Ethiopia, from January 2018 to June 2018 (Figure 1). Arba Minch Zuria district is situated in the well-known areas of East Africa rift valley and surrounded by lakes Abaya and Chamo, as well as the Nech Sar National Park (NSNP). The district is bordered to the South by Derashe special district, North by Chencha and Dita districts, on the West by Bonke district, on the Northeast by Mirab Abaya, and on the Southeast by Amaro special districts of Southern Ethiopia. Topographically, plains, massifs, gorges, and steep slopes along the course of a number of rivers and streams mark these areas. The altitude of the district ranges from 1001 to 2500 masl. The area has a bimodal rainfall pattern, with a long rainy season between June to September, and the short rain falls between March and April. Annual rainfall ranges from 800 to 1200 mm, and the average annual temperature is 26.33°C. The district is divided into midland and lowland agro-ecological zones, which account for about 45.5% and 55.55% of the total area, respectively. The total cattle population in the district is estimated to be 155,617. The livelihood of the society largely depends on mixed crop and livestock production system [1, 23]. Also, the study area is highly infested with *G. pallidipes* and biting flies like *Stomoxys* and *Tabanus* [13, 15, 16]. Tsetse flies to be examined in the present study were caught from two kebeles of the district, namely, Ganta Kanchama and Kola shara, and Nech Sar National Park, which is one of highly tsetse-infested areas in the country.

### 2.2. Study Design

A cross-sectional study design was employed in the Arba Minch Zuria district of Gamo Zone, Southern Ethiopia, to determine population density and trypanosome infection rate of *G. pallidipes.*

### 2.3. Study Methodology

#### 2.3.1. Entomological Study

For this entomological study, purposive sampling was applied based on the complaint by animal owners on tsetse-transmitted bovine trypanosomosis to select two study kebeles from the district and nearby Nech Sar National Park of Southern Ethiopia. In Ethiopia, kebele refers to the lowest administrative division of district but greater than village. The selected two kebeles were Ganta Kanchama and Kola Shara. A total of 40 standard NGU traps were deployed around the watering and grazing areas for trapping of tsetse and other biting flies in the study area. NGU trap is used to catch tsetse flies and is very effective and easily constructed from locally available material [24]. To attract the flies, all traps were uniformly baited with acetone and 3-week-old cow urine and deployed at an interval of 200 meters a part [25]. Traps were allowed to stay at the site of deployment for a maximum period of 72 hours before collection [26]. Trap deployment sites were selected to represent all habitat or vegetation types in the study area that could be associated with tsetse fly feeding, behavior, multiplication, and other related aspects. The poles of each trap were carefully greased to prevent tsetse fly predators mainly ants. Then, tsetse and other biting flies trapped were collected and counted [26, 27].

Tsetse flies were sexed by observing the posterior end of the ventral aspect of the abdomen using a hand lens and stereomicroscope. Hence, male flies were identified by an enlarged hypopygium in the posterior ventral part of the abdomen, which is absent in female flies. Other caught biting flies were identified to genera level according to their morphological characteristics such as size, color, wing venation structure, and proboscis [26–28]. The apparent density of the tsetse fly was calculated as the number of tsetse catch/trap/day [29].

#### 2.3.2. Tsetse Fly Dissection

Freshly collected tsetse flies were immediately subjected to dissection and examination for infection with trypanosome species [27]. The dissection procedure was carried out as described in the FAO Training manual for tsetse control personnel [26]. Firstly, the wings and legs of tsetse flies were removed. Then, wing fray and ovary analysis was performed to determine the age of male and female tsetse flies, respectively. 0.95% normal saline solution was used for dissecting freshly killed tsetse flies under a dissecting microscope [26, 30].

Three body parts of tsetse flies, namely, proboscis (mouth part), midgut, and salivary glands, were examined. A compound microscope at a magnification of ×400 times was used for identification of trypanosome infections in the tsetse flies [28]. Trypanosome parasites detected in the mouthpart only were considered in the group of subgenus Duttonella (*T. vivax* and *T. uniformis*), those detected in both the mouthparts and midguts were considered in the group subgenus Nanomonas (*T. congolense* and *T. simiae*), and those found in midgut, salivary glands, and mouthparts were considered Trypanozoon (*T. brucei brucei*, *T. brucei gambiense*, and *T. brucei rhodesiense*). Infections were considered immature when the trypanosome parasites were detected only in the midgut of tsetse flies. Finally, Giemsa-stained smears were examined under an oil immersion compound microscope (100 times magnification) for trypanosome species identification based on their morphological appearances [27, 30, 31].

#### 2.3.3. Infection Rate Determination

The trypanosome infection rate (IR) of tsetse flies was calculated using the following formula [32]:

(1) $$\begin{array}{c} \text{Infection rate }\left(\text{IR}\right)=\frac{\text{Number of tsetse flies infected}}{\text{Total number of tsetse flies dissected over a given period}}. \end{array}$$

### 2.4. Data Management and Statistical Analysis

Entomological data collected from each deployed trap were coded and recorded in Microsoft excel, 2010 spread sheet. STATA version 14 computer software was applied for the statistical analysis at 95% confidence interval. The infection rates (IR) of trypanosomes in *G. pallidipes* were calculated as the number of microscopically positive flies divided by the total number of dissected flies and multiplied by 100. The apparent density of tsetse and biting flies was expressed as the number of each type of flies per traps per day (FTD). Trypanosome infection in *G. pallidipes* and its association with potential risk factors were computed using univariable logistic regression analysis. In all cases, 95% of confidence intervals were used, and *p* value less than 0.05 was considered significant [33].

## 3. Results

### 3.1. Proportions of Each Fly Count

From 40 standard NGU traps deployed during the study period, a total of 2176 flies were caught (Table 1). Of these, 1803 (82.86%) belong to tsetse flies, and the remaining 373 (17.14%) were biting flies. *G. pallidipes* was identified as the only tsetse fly species in the study area. The biting flies that are commonly encountered during the study period were genera *Tabanus* (53.35%) and *Stomoxys* (46.65%).

### 3.2. Distribution and Abundance of *Glossina pallidipes* and Other Biting Flies

The overall apparent densities of *G. pallidipes* and other biting flies in the study area were 15.03 F/T/D (fly/trap/day) and 3.11 F/T/D, respectively (Table 2). High apparent density of tsetse and biting flies was found in Nech Sar National Park (4.67 F/T/D) compared with domestic animal grazing areas of Ganta Kanchama (1.77 F/T/D) and Kola Shara kebeles (1.33 F/T/D).

**Figure 2:**
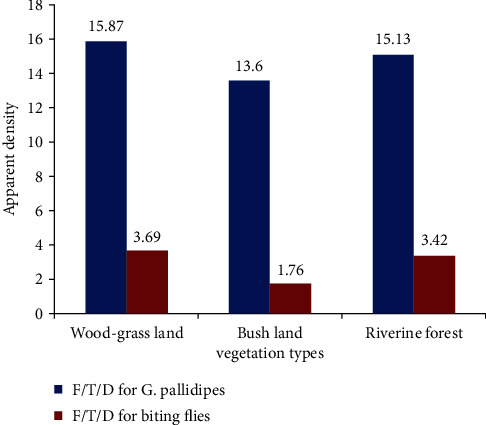
Apparent density based on vegetation types in the study area.

The different habitats of vegetation were assessed during entomological survey period, and there was a variation in apparent density distribution of tsetse and biting flies in three vegetation types in the study area (Figure 2). Relatively peak infestation of *G. pallidipes* and biting flies was observed in wood-grass land (WGL) than riverine forest and bush land vegetation types. The apparent density for tsetse flies was 15.87, 15.13, and 13.6, respectively, for wood-grass land, riverine forest, and bush land vegetation types during the study period, while the apparent density for biting flies was 3.69, 3.42, and 1.76, respectively, for wood-grass land, riverine forest, and bush land vegetation types in the study area.

### 3.3. Trypanosome Infection Rate in *Glossina pallidipes*

From a total of 300 dissected *G. pallidipes*, 53 flies were infected with trypanosomes resulting in an overall infection rate of 17.67% in the study area (Table 3). High-trypanosome infections were observed in the Nech Sar National Park (26%) followed by Kola Shara (11.42%) and Ganta Kanchama (7.5%) kebeles of Arbaminch Zuria district. *T. congolense* (66.04%) was the predominant species and found to be a major cause of tsetse fly infection in the study area followed by *T. vivax* (33.96%). Infection of *G. pallidipes* due *T. brucei* and mixed type of infection was not found in the study area during study period.

### 3.4. Analysis of *Glossina pallidipes* Infection with Possible Risk Factors

Trypanosome infection in *G. pallidipes* and its association with potential risk factors were summarized in univariable logistic regression analysis (Table 4). This result showed that trypanosome infection in female *G. pallidipes* was 3.39 significantly higher than that in male *G. pallidipes*. There was a strong significant difference (*p* < 0.05) between age-related effects in trypanosome infections of *G. pallidipes*. Trypanosome infection in flies older than 20 days was 2.5 times higher than that in those aged less than 20 days. Also, there was variation in trypanosome infection between different hunger stages (nutritional) of collected tsetse flies. The odds of getting trypanosome infection in stage 1 flies are 14.24 times higher than those in stage 4 flies. Trypanosome infection in and around park grazing area was 3.41 times higher than in communal grazing areas. Also, the odd of getting trypanosome infection in *G. pallidipes* in wood-grass land was 2.7 times higher than that in bush land vegetation type.

## 4. Discussion

This study was conducted to estimate the infection rate of trypanosome in *G. pallidipes*, their distribution, and the trypanosome species involved in Arbaminch Zuria district of Gamo zone, Southern Ethiopia. This kind of study is useful for implementation of appropriate methods for control and suppression of the disease and its vector in the study area. The study revealed the presence of only one Glossina species, *G. pallidipes*, and other biting flies including *Stomoxys* and *Tabanus* in the study area. A total of 1803 (82.86%) *G. pallidipes* and 373 (17.14%) other biting flies were caught during the study period. The overall apparent density of *G. pallidipes* and biting flies in the study area was 15.03 F/T/D (fly/trap/day) and 3.11 F/T/D, respectively, which is low when compared with previous reports by Teka et al. [18] and Rodrigues et al. [16] who found an overall apparent density of *G. pallidipes* and biting flies as 29.624 F/T/D and 47.8 F/T/D, respectively, in the study area. These differences might be due to variations in study seasons and tsetse control strategies applied in each study year in the study area. However, reports by Yalew and Fantahun [34], Desta et al. [35], Abebe et al. [4], Eyasu et al. [14], Anjulo et al. [17], and Meharenet and Alemu [20] in different parts of Ethiopia showed a lower apparent density in comparison to the present study. These variations are probably due to differences in vegetation types, availability of domestic and wild host animals, and study seasons and tsetse control strategies applied in each of respective study sites [21, 25, 36].

There was variation in apparent density of tsetse and biting flies in the three vegetation types available in the study area. Relatively higher *G. pallidipes* and biting flies were caught in wood-grass land (15.87F/T/D) and riverine forest (15.13F/T/D) than bush land (13.6 F/T/D) vegetation types. In a close agreement to this result, Desta et al. [35] in Birbir valleys of Western Ethiopia and Dagnachew et al. [37] in the Blue Nile basin areas of Northwest Ethiopia showed high apparent density of tsetse flies in the riverine and savanna woodland vegetation types followed by forest, bush, and cultivated areas. Also, similar findings were reported by Ouma et al. [38], Nthiwa et al. [39], and Cecchi et al. [40] in other eastern African countries such as Uganda, Kenya, and Somalia, respectively. This is because open savanna woodland and riverine forest edges are typical habitat for *Glossina* morsitans group (mainly for *G. pallidipes*) [32, 41–43].

From a total of 300 dissected *G. pallidipes*, 53 flies were infected with trypanosome resulting in an overall infection rate of 17.67% in the study area. Compared to the present finding, reports done by Rodrigues et al. [16] showed high trypanosome infection rate (38%) in *G. pallidipes* inside Nech Sar National Park of Arba Minch Zuria district, Southern Ethiopia. According to Desta et al. [31] and Meharenet and Alemu [20], a relatively low fly infection rate was observed in Amaro special district of Southern Ethiopia and Limmu Kosa district of Jimma zone, Western Ethiopia, respectively. Also, lower infection rates have been reported by Nthiwa et al. [39] who indicated 11.53% from Mouhoun river in Burkina Faso, Bouyer et al. [44] who reported 10% from Mtito Andei Division in Kenya, Nnko et al. [21] who reported 5.8% from Maasai Steppe in Tanzania, and Kame-Ngasse et al. [45] who reported 0.9% from Adamawa region in Cameroon. This might be due to least tsetse challenge, variation in Glossina species involved, and low fly animal contact during the study period [46].

Two species of trypanosomes were identified and found to be a major cause of tsetse fly infection in the study area, namely, *T. congolense* (66.04%) and *T. vivax* (33.94%). Likewise, different authors across Africa reported *T. congolense* as leading species for tsetse fly infection [39, 44, 45]. Moreover, Abebe and Jobre [8] and Langridge [30] stated *T. congolense* as one of the important mouthparts and midgut trypanosome parasites because of its pathogenicity to cattle and its relatively higher infection rate in *G. pallidipes* which was completely supported by the present findings.

The univariable regression analysis for potential risk factors showed that significantly higher trypanosome infections were observed in female (OR = 3.39, CI = 1.53‐7.52), older age group (OR = 2.5, CI = 1.12‐5.56), and hunger stage one (OR = 14.24, CI = 4.01‐50.59) of *G. pallidipes*, wood-grass land vegetation type (OR = 2.7, CI = 1.2 − 6.5), and in nearby park grazing areas (OR = 3.41, CI = 1.76‐6.6) of the study district. More trypanosome infections were observed in female *G. pallidipes* (22.61%) than in male *G. pallidipes* (7.92%). A similar finding was reported by Bitew et al. [47] in Gojeb valley of Southwest Ethiopia, Desta et al. [31] nearby Amaro special district of Southern Ethiopia, and Meharenet and Alemu [20] in Limmu Kosa districts of western Ethiopia. The reason for a comparatively higher trypanosome infection rate in female *G. pallidipes* might be due to their better life expectancy, and lower infection rate found in male *G. pallidipes* flies can be explained by the low average age of trapped male flies (20 days or less) as suggested by different authors [20, 31, 41]. Also, it is because of the female tsetse flies physiologically necessitated to feed more animal blood from many animals during their pregnancy than male's tsetse flies which exposes them for high infection rate [48].

Based on a microscopic examination for the contents of the uterus and wing fray analysis, a higher trypanosome infection rate was observed in *Glossina pallidipes* older than the 20-day age group (20.83%) than the below 20-day age group (9.52%). This result was in a close agreement with the previous study reported by Meharenet and Alemu [20] and Desta et al. [31]. This is because an older fly will have more chance to become infected and also have more time for its infection to become mature [32, 49].

In the current study, there was variation in trypanosome infection between different hunger stages (nutritional) of collected tsetse flies. Accordingly, flies in stage 1 (47.62%) and stage 2 (14.43%) were highly susceptible than flies in stage 3 (6.67%) and stage 4 (6%). In close agreement to this, Meharenet and Alemu [20] reported higher trypanosome infection in stage 1 (gorged) and stage 2 (replete) tsetse flies in Loma district of Southern Ethiopia. It is because under natural conditions, nutritional stress (hunger stage) in tsetse flies could contribute to substantial increase in trypanosome infection rate which was completely supported by the present findings [32, 49].

Trypanosome infection rate of *G. pallidipes* in and around park grazing area (26%) was significantly (*p* < 0.05) higher than that in and around communal (domestic animal) grazing areas (9.33%). The presence of high trypanosome infection rate in park grazing areas could be associated with factors such as availability of different vegetation types like bush land and riverine forest which create a suitable condition for growth and development of tsetse flies and presence of many wild animals as trypanosome reservoirs in areas close to the park which intern increases contact with vectors and controls programs applied [21].

## 5. Conclusion

This study presents findings on the trypanosome infection rate of *G. pallidipes*, their apparent density, and involved trypanosome species in Arbaminch Zuria districts of Southern Ethiopia. The entomological findings revealed the presence of only one Glossina species, known as *G. pallidipes*, and other biting flies including *Stomoxys* and *Tabanus*. The overall apparent density of *G. pallidipes* and biting flies in the study area was 15.03 F/T/D and 3.11 F/T/D, respectively. Relatively higher *G. pallidipes* and biting flies were caught in wood-grass land and riverine forest than bush land vegetation types. The overall trypanosome infection rate of *G. pallidipes* was 17.67%. Two trypanosome species, namely, *T. congolense* and *T. vivax*, were responsible for *G. pallidipes* infection in the study area. Trypanosome infection rate was significantly higher in female, older age group, and hunger stage 1 *G. pallidipes*, woody grassland vegetation type, and in nearby park grazing area of the study district. Moreover, this study showed high apparent density of *G. pallidipes* and its infection by trypanosome. Therefore, this study warrants the need for strengthening both the vector and parasite control interventions in the current study area.

### 5.1. Limitation

The study could not include trypanosome subspecies and blood-feeding preference of involved vector (*G. pallidipes*) which can be distinguished only by isoenzymatic differences and molecular techniques such as polymerase chain reaction (PCR).

## Figures and Tables

**Table 1:** Proportions of each fly count in the study area.

Kebele	Tsetse fly caught (%)			Biting flies caught (%)			Total fly count
	Male	Female	Total	*Tabanus*	*Stomoxys*	Total	
Ganta Kanchama	95 (23.57)	308 (76.43)	403	27 (50.94)	26 (49.06)	53	456
Kola Shara	70 (38.9)	110 (61.1)	180	24 (0.6)	16 (0.4)	40	220
Nech Sar Park	426 (34.92)	794 (65.08)	1220	167 (59.64)	113 (40.36)	280	1500
Total	591 (32.78)	1212 (67.22)	1803	199 (53.35)	174 (46.65)	373	2176

**Table 2:** Distribution and abundance of *G. pallidipes* and other biting flies in the study area.

Variable	Category	Traps deployed	*G. pallidipes*			Biting flies		
			Male	Female	Total	F/T/D	Count	F/T/D
Kebele	Ganta Kanchama	10	95	308	403	13.4	53	1.77
	Kola Shara	10	70	110	180	6	40	1.33
	Nech Sar Park	20	426	794	1220	20.33	280	4.67
	Total	40	591	1212	1803	15.03	373	3.11

Vegetation type	Wood-grass land	15	381	333	714	15.87	166	3.69
	Bush land	10	100	308	408	13.6	53	1.76
	Riverine forest	15	110	571	681	15.13	154	3.42
	Total	40	591	1212	1803	15.03	373	3.11

F/T/D: fly/trap/day.

**Table 3:** Trypanosome infection rate in *G. pallidipes* and identified trypanosome species.

Kebele	Number of flies dissected	Number of infected flies (%)	*T. congolense* (%)	*T. vivax* (%)	Overall infection (%)
Kola Shara	70	8 (11.42)	6 (75)	2 (25)	8 (11.42)
Ganta Kanchama	80	6 (7.5)	2 (33.33)	4 (66.66)	6 (7.5)
Nech Sar Park	150	39 (26)	27 (69.23)	12 (30.76)	39 (26)
Total	300	53 (17.67)	35 (66.04)	18 (33.94)	53 (17.67)

**Table 4:** Univariable logistic regression analysis for potential risk factors of trypanosome infection in *G. pallidipes.*

Variable	Category	No. of fly examined	No. of fly infected	Infection rate (%)	OR	95% CI	*p* value
Sex	Male	101	8	7.92	—	—	Ref
	Female	199	45	22.61	3.39	1.53-7.52	0.003

Age	≤20 days	84	8	9.52	—	—	Ref
	>20 days	216	45	20.83	2.5	1.12-5.56	0.025

Hunger stage	Stage 4 (hungry)	50	3	6	—	—	Ref
	Stage 3 (intermediate)	90	6	6.67	1.11	0.26-4.68	0.87
	Stage 2 (replete)	97	14	14.43	2.64	0.72-9.67	0.14
	Stage 1 (gorged)	63	30	47.62	14.24	4.01-50.59	0.001

Grazing area	Communal/domestic	150	14	9.33	—	—	Ref
	Wild/park area	150	39	26	3.41	1.76-6.60	0.01

Vegetation type	Bush land	85	8	9.41	—	—	Ref
	Riverine forest	95	18	18.94	2.25	0.74-5.48	0.07

Kebele	Wood-grass land	120	27	22.5	2.7	1.2-6.5	0.017
	Ganta Kanchama	80	6	7.5	—	—	Ref
	Kola Shara	70	8	11.42	1.59	0.52-4.83	0.41
	Nech Sar Park	150	39	26	4.33	1.74-10.74	0.002

## Data Availability

The raw data supporting the result and conclusions of this article will be made available by the authors, without undue reservation.

## Abbreviations

FTD: Fly/trap/day
GDP: Gross domestic product
IR: Infection rate
*G. pallidipes*: *Glossina pallidipes*
*T. brucei*: *Trypanosoma brucei*
*T. vivax*: *Trypanosome vivax*
*T. congolense*: *Trypanosome congolense*
masl: Meter above sea level
OR: Odds ratio.
